# Recalcitrant Palmar Warts Treated With Q-Switched Potassium Titanyl Phosphate Laser and Cryotherapy: A Case Report

**DOI:** 10.7759/cureus.89605

**Published:** 2025-08-08

**Authors:** Virgilio Blandon, Ruby Poveda, Krimhild Serrano

**Affiliations:** 1 Dermatology, Hospital Carlos Roberto Huembes, Managua, NIC

**Keywords:** non-ablative laser, palmar warts, potassium titanyl phosphate laser, q-switched laser, recalcitrant warts

## Abstract

Recalcitrant palmar common warts pose a considerable challenge in dermatology due to their frequent persistence despite various treatment attempts. The thick stratum corneum of the palms and the constant pressure and friction in this location contribute to their resistance to therapy and a high rate of recurrence. We report the case of a 33-year-old male with a 26-month history of a progressively enlarging palmar wart refractory to extensive conventional therapies, including 18 intermittent sessions of liquid nitrogen cryotherapy administered over the course of his 26-month history, two electrofulguration sessions, and various topical agents. We applied a novel multimodal treatment involving superficial shaving, followed by Q-switched potassium titanyl phosphate laser at 12 J/cm² fluence, combined with cryotherapy using liquid nitrogen. After two sessions, administered two weeks apart, complete clinical and dermatoscopic resolution was achieved within two weeks of the final treatment. No recurrence was observed at a three-month follow-up. The patient experienced minimal adverse effects, with mild and transient discomfort and erythema, but no vesiculation, blistering, or pain. Our literature search revealed no previously reported cases of recalcitrant palmar verruca vulgaris successfully treated with Q-switched potassium titanyl phosphate laser using this multimodal protocol. This case highlights a promising and previously undescribed combined strategy for managing highly refractory palmar warts unresponsive to standard treatments.

## Introduction

Common warts, medically known as verruca vulgaris, are benign skin growths caused by infection with specific human papillomavirus (HPV) types, most commonly HPV 2, 4, and 7 in palmar and plantar locations [1-2]. These lesions are commonly diagnosed and frequently affect the hands and feet, where viral inoculation often occurs due to trauma [3,4]. The estimated prevalence of common warts ranges from 22% to 33% [5,6]. Many warts resolve spontaneously over months to years, with approximately 66% disappearing within two years [7]; however, a significant proportion become resistant to treatment [8,9]. In adults, warts can be particularly persistent, sometimes requiring five to 10 years to achieve clearance [10]. The incidence of palmar warts specifically has been reported to range from 4.5% to 29.4%, highlighting their relatively frequent presentation in this anatomical site [3,4].

Cutaneous warts manifest in various forms across different body sites, and treatment is often sought due to pain, bleeding, contagiousness, or cosmetic concerns [11]. In fact, a study on the morbidity of cutaneous warts found that a significant proportion of subjects reported physical discomfort and a moderate to extreme impact on social or leisure activities, highlighting the substantial burden of these lesions on patients' quality of life [11]. Recalcitrant palmar verruca vulgaris, in particular, represents a considerable therapeutic challenge in dermatology, frequently persisting despite diverse treatment approaches [8]. The medical literature describes numerous therapeutic options, including monotherapies and combination therapies [2,9,12,13]. However, topical keratolytic agents often demonstrate limited efficacy, and cryotherapy or surgical ablation methods can be painful and not universally successful [2,11]. Furthermore, few available treatments have been evaluated through rigorous, blinded, randomized controlled trials [13]. The histopathology of common warts is characterized by acanthosis, significant hyperkeratosis, and papillomatosis, often forming a "church spire" pattern [12]. This papillomatosis is associated with increased vascularization, which provides the blood supply necessary for lesion growth [12]. Consequently, laser therapies have been investigated as alternative options, with different modalities targeting distinct chromophores [14]. Some lasers, like the CO₂ and Er:YAG, function through direct tissue ablation by targeting water, while others, such as the pulsed dye laser (PDL) and neodymium-doped yttrium aluminum garnet (Nd:YAG), target oxyhemoglobin to destroy the wart's microvasculature [14].

The efficacy of potassium titanyl phosphate (KTP) laser for verrucae has been explored in limited studies [8,14,15]. A retrospective analysis of 29 patients reported 82.8% complete recovery using a long-pulsed KTP laser, while another study demonstrated 80% improvement in 25 recalcitrant cases treated with continuous wave KTP laser [8,15]. The KTP laser operates at a 532 nm wavelength, selectively targeting oxyhemoglobin in vascular structures [8]. Although both long-pulsed and Q-switched KTP lasers utilize this wavelength, their distinct pulse durations produce different mechanisms of action [8,14-17]. Long-pulsed KTP lasers deliver energy over milliseconds, inducing photothermal coagulation of blood vessels, a mechanism commonly used to treat vascular lesions [8,15,17]. In contrast, Q-switched KTP lasers, like the one used in our case, deliver 10-nanosecond pulses that generate ultra-short energy bursts [16,18]. This rapid energy delivery creates a photoacoustic effect, resulting in mechanical disruption of targeted microvasculature, a mechanism employed in the treatment of both pigmentary and certain vascular lesions [16,18]. However, given that recalcitrant warts are highly vascularized, the disruption of their blood supply is a key therapeutic strategy, making the Q-switched KTP laser a plausible modality for their treatment [14,15,17-19].

Despite the availability of diverse treatment options, recalcitrant palmar verruca vulgaris remains a significant therapeutic challenge [8,9]. While the long-pulsed KTP laser has shown promising efficacy [8,14,16], our literature search identified no prior reports of Q-switched potassium titanyl phosphate laser being used for verruca vulgaris, either as monotherapy or as part of a multimodal protocol. This case report describes a previously unreported combined approach using a Q-switched potassium titanyl phosphate laser combined with cryotherapy for managing highly recalcitrant palmar warts unresponsive to conventional treatments. Given its observed efficacy, this laser-based multimodal approach may represent a valuable therapeutic option in clinical settings where a Q-switched KTP laser is available but other conventional laser therapies for warts are not.

## Case presentation

A 33-year-old male physician, with an unremarkable past medical history and no known immunocompromising conditions, presented with a 26-month history of a progressively enlarging verruca vulgaris on the right palm. The lesion had been previously treated by three different dermatologists using a variety of conventional therapies over a two-year period. The patient underwent 18 sessions of cryotherapy with liquid nitrogen, two electrofulguration treatments, and various topical medications. The topical regimens involved 20% salicylic acid lotion, a glycyrrhizinic acid-based preparation applied for six months, and imiquimod 5% cream used for two months. Despite these efforts, the lesion showed no clinical improvement. Notably, the patient had also developed scarring on the palm as a consequence of repeated interventions.

**Figure 1:**
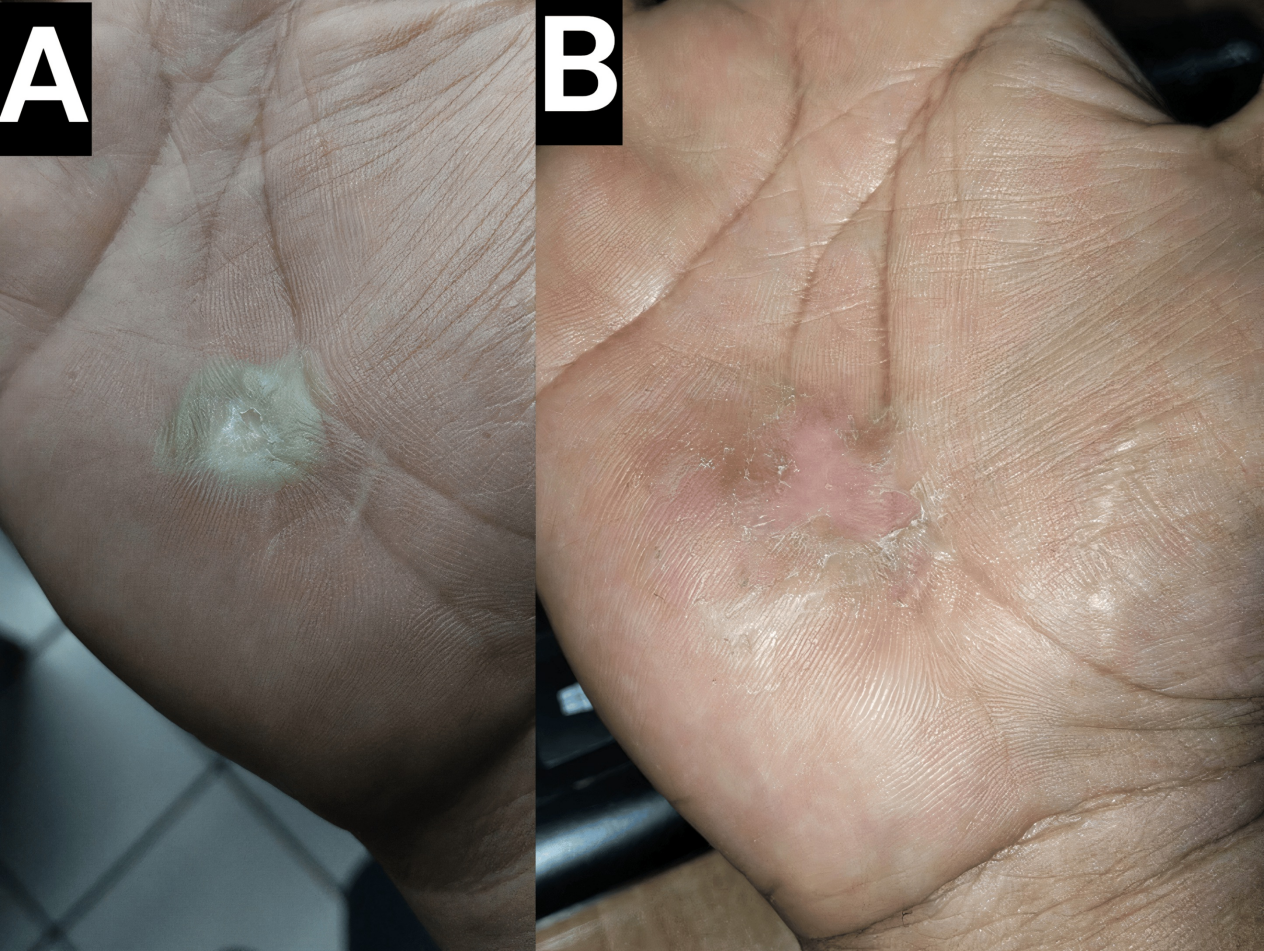
Clinical presentation of the palmar wart (A) Baseline clinical image of the verruca vulgaris on the patient's right palm before treatment initiation. (B) Clinical image 24 hours after the first combined treatment session with superficial shaving, Q-switched KTP laser, and cryotherapy, showing significant early improvement.

Physical examination revealed a 1.5 cm, round, raised, hyperkeratotic, whitish, opaque, and rough papule on the distal aspect of the right thenar eminence, bordering the central palm (Figure 1A). Dermatoscopic evaluation demonstrated characteristic dotted and linear hemorrhagic points within the lesion, consistent with verruca vulgaris [19]. The patient verbally reported mild discomfort and functional inconvenience, describing pain on gripping objects and during writing. In view of the lesion’s resistance to standard therapies, a multimodal treatment strategy was initiated.

**Figure 2:**
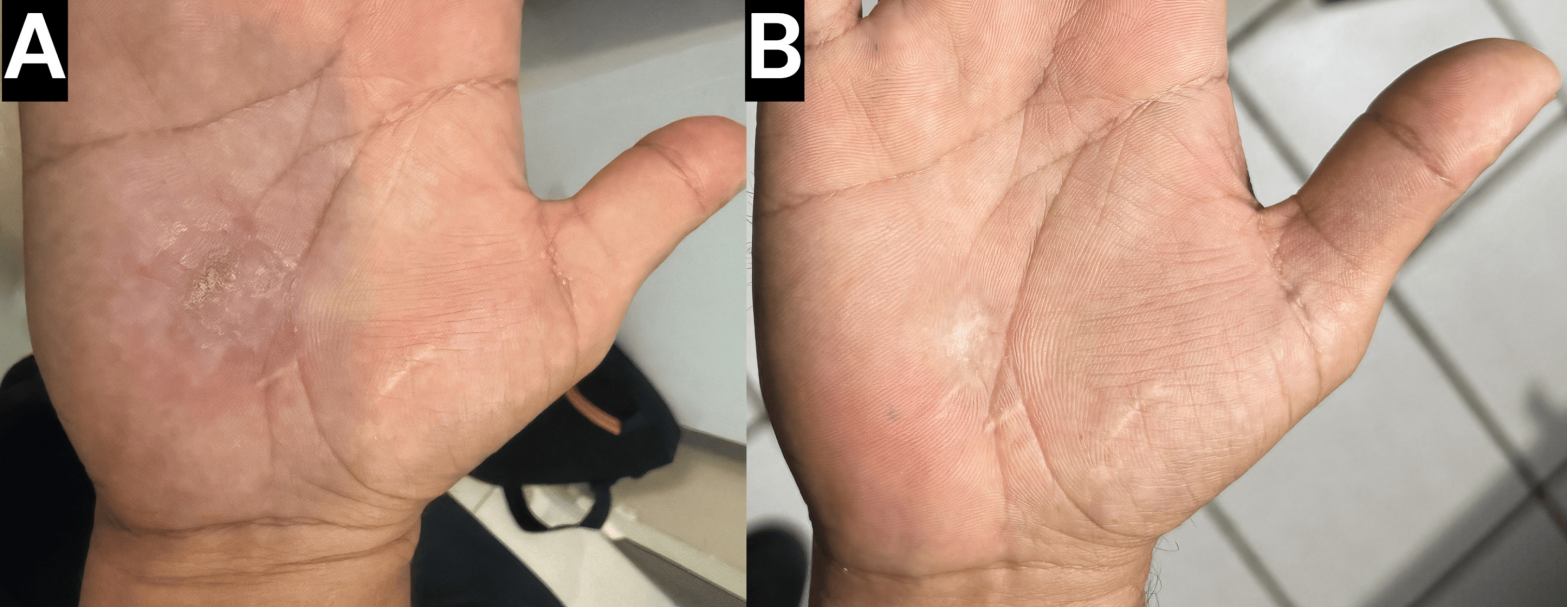
Clinical evolution following combined therapy (A) Clinical image of the right palm prior to the second treatment session (2 weeks after the first session), showing continued improvement. (B) Clinical image at the 3-month follow-up after the second treatment session, demonstrating complete clinical resolution of the verruca vulgaris with no signs of recurrence.

During the initial treatment session, the hyperkeratotic surface of the wart was gently debulked using superficial shaving. This was immediately followed by the application of a Q-switched KTP laser (532 nm wavelength, 10-nanosecond pulse duration, 3 mm spot size, 12 J/cm² fluence, five passes). The laser was used to target the lesion’s vascular component through photoacoustic disruption of the microvasculature [16]. Finally, liquid nitrogen cryotherapy was applied using an open-spray technique for three seconds to achieve a 2 mm ice halo, and this was repeated for three freeze-thaw cycles. On follow-up 24 hours later, clinical assessment revealed a visible reduction in lesion bulk and vascularity (Figure 1B). Two weeks after the first session, the patient returned for a second treatment session, at which point continued improvement was observed and the same multimodal protocol (superficial shaving, Q-switched KTP laser, and cryotherapy) was repeated (Figure 2A).

**Figure 3:**
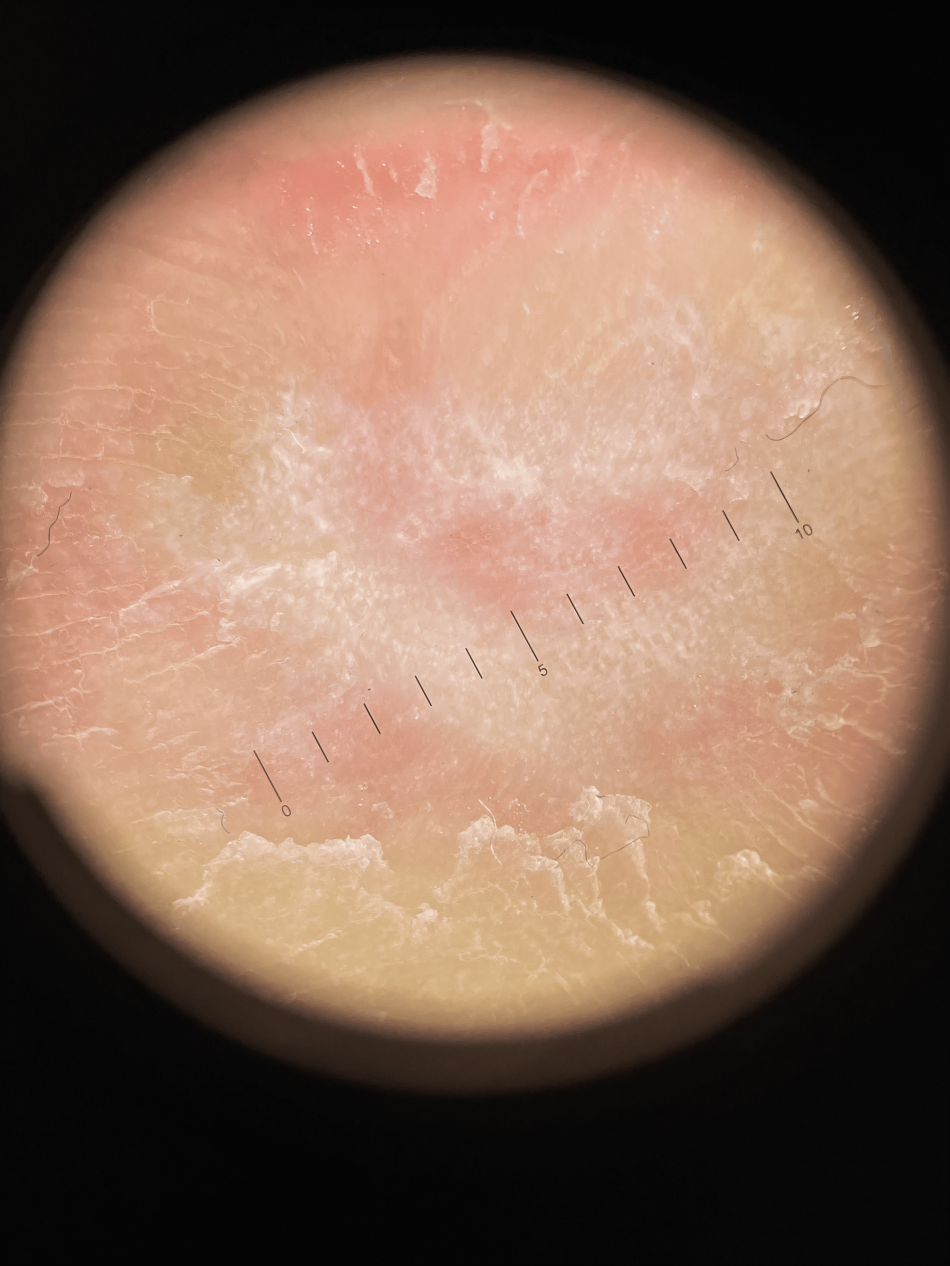
Dermatoscopic examination at three-month follow-up Dermatoscopic image of the treated area on the right palm at the three-month follow-up, showing complete absence of characteristic verruca vulgaris vascular patterns, indicative of clinical and subclinical resolution.

At the three-month follow-up visit after the second session, the lesion, which measured 1.5 cm at baseline, had completely resolved with no evidence of recurrence (Figure 2B). The patient tolerated the procedure well and reported minimal adverse effects, with mild and transient discomfort and erythema, but no vesiculation, blistering, or pain. Dermatoscopic evaluation at this visit also confirmed the absence of any dotted or linear hemorrhagic points, indicating complete resolution (Figure 3).

## Discussion

The significant clinical improvement seen in our patient, who had a longstanding palmar wart resistant to several standard treatments, highlights the ongoing difficulty in managing recalcitrant warts and the necessity for new treatment options [8]. This case demonstrates a successful multimodal approach that resulted in complete resolution within three months, with only mild and transient adverse effects, such as erythema and discomfort.

Our literature review revealed no prior reports of Q-switched potassium titanyl phosphate (KTP) laser being used for verruca vulgaris, either as monotherapy or in combination therapy. On July 18, 2025, systematic searches were conducted across PubMed, DOAJ, Google Scholar, ClinicalTrials.gov, and SciELO. The PubMed search included terms such as (warts OR verruca* OR “common wart” OR “palmar wart” OR “plantar wart”) AND (“KTP laser” OR “potassium titanyl phosphate laser” OR “Q-switched KTP laser” OR “Q-switched laser”) AND (treatment OR therapy OR management), yielding five results, none of which involved Q-switched KTP lasers. Similar queries across DOAJ and ClinicalTrials.gov yielded zero results, while Google Scholar returned one non-relevant article. SciELO searches using comparable terms also yielded no relevant findings. This supports the novelty of our approach.

Existing literature on KTP lasers for wart treatment primarily involves long-pulsed or continuous wave modes [8,15]. Continuous wave lasers typically induce photothermal coagulation, while long-pulsed modes operate with millisecond pulse durations to achieve selective photothermolysis [8,15]. In contrast, the Q-switched KTP laser (532 nm, 10-nanosecond pulse duration, 3-mm spot size, 12 J/cm² fluence, 5 passes), as used in our case, delivers energy in ultra-short bursts, creating a photoacoustic effect that leads to mechanical disruption of the microvasculature rather than thermal damage [18]. This mechanism is well-documented in treating superficial vascular lesions such as telangiectasias and reticular veins [18], and may have contributed significantly to wart resolution in this case by disrupting the lesion’s blood supply.

Initial debulking through superficial shaving reduced the hyperkeratotic barrier, a critical step for enhancing laser penetration, given the limited dermal depth reached by 532 nm wavelengths (~0.8 mm) [17]. Subsequent Q-switched KTP application likely compromised the vascular supply, weakening the lesion. Finally, cryotherapy via open-spray technique (3-second sprays, 3-mm ice halo, three freeze-thaw cycles) induced necrosis of virally infected keratinocytes and further vascular disruption [20]. Combined, these treatment modalities appear to have been effective, resulting in full clinical resolution. This case suggests that a multimodal approach incorporating a Q-switched KTP laser may offer a promising therapeutic option for treatment-resistant palmar verruca vulgaris, particularly in patients with a history of failure with conventional therapies. The excellent cosmetic outcome and absence of significant adverse effects support its potential utility.

However, several limitations must be acknowledged. As a single case report, the findings cannot be generalized, and further research is required to validate efficacy. Although the diagnosis was strongly supported by characteristic clinical and dermoscopic features, histological confirmation was not obtained. This was a deliberate clinical decision made to avoid further trauma to the patient, given the history of repeated interventions and the accepted practice of diagnosing verruca vulgaris based on clinical features. Furthermore, the specific HPV type was not identified due to a lack of resources at our institution, which could have provided valuable insights into the wart's etiology and its recalcitrance to prior therapies. Additionally, the photographic documentation lacks scale markers, a limitation due to the case not being initially designed for publication. Future comparative studies are necessary to determine the individual contribution of each modality, assess recurrence rates, and define optimal parameters for treatment, all of which should incorporate definitive diagnostic methods to strengthen the findings.

## Conclusions

This case suggests that a multimodal approach, including the Q-switched KTP laser, may serve as an effective option for treating resistant palmar common warts, particularly in patients who have failed conventional treatments. The observed complete resolution, excellent cosmetic outcome, and good patient tolerance suggest a potential clinical benefit in settings where a Q-switched KTP laser is available but other conventional laser therapies for warts are restricted.

Nonetheless, given the limitations inherent to a single case report, these findings should be interpreted with caution. Further research is necessary to confirm efficacy, define the role of each treatment modality, and establish optimal parameters and recurrence rates over time. To guide future research, we suggest controlled studies with larger patient cohorts, such as randomized trials comparing our multimodal approach with standard monotherapies (e.g., cryotherapy alone) or other combination therapies. Such studies would also be essential for determining the individual contribution of each modality to the final outcome.
